# Low income, community poverty and risk of end stage renal disease

**DOI:** 10.1186/1471-2369-15-192

**Published:** 2014-12-04

**Authors:** Deidra C Crews, Orlando M Gutiérrez, Stacey A Fedewa, Jean-Christophe Luthi, David Shoham, Suzanne E Judd, Neil R Powe, William M McClellan

**Affiliations:** Division of Nephrology, Department of Medicine, Johns Hopkins Medical Institutions, 301 Mason F. Lord Drive, Suite 2500, Baltimore, MD 21224 USA; Welch Center for Prevention, Epidemiology and Clinical Research, Johns Hopkins Medical Institutions, Baltimore, MD USA; Division of Nephrology, Department of Medicine, University of Alabama Birmingham, Birmingham, AL USA; Department of Epidemiology, University of Alabama Birmingham, Birmingham, AL USA; Department of Epidemiology, Emory University, Atlanta, GA USA; Institute of Social and Preventive Medicine, Centre Hospitalier Universitaire Vaudois and University of Lausanne, Lausanne, Switzerland; Department of Public Health Sciences, Loyola University Chicago, Maywood, IL USA; Department of Biostatistics, University of Alabama Birmingham, Birmingham, AL USA; Department of Medicine, San Francisco General Hospital, San Francisco, CA USA; Department of Medicine, University of California at San Francisco, San Francisco, CA USA; Department of Medicine, Emory University, Atlanta, GA USA

**Keywords:** ESRD, Chronic kidney disease, Socioeconomic status, Disparity, Geospatial

## Abstract

**Background:**

The risk of end stage renal disease (ESRD) is increased among individuals with low income and in low income communities. However, few studies have examined the relation of both individual and community socioeconomic status (SES) with incident ESRD.

**Methods:**

Among 23,314 U.S. adults in the population-based Reasons for Geographic and Racial Differences in Stroke study, we assessed participant differences across geospatially-linked categories of county poverty [outlier poverty, extremely high poverty, very high poverty, high poverty, neither (reference), high affluence and outlier affluence]. Multivariable Cox proportional hazards models were used to examine associations of annual household income and geospatially-linked county poverty measures with incident ESRD, while accounting for death as a competing event using the Fine and Gray method.

**Results:**

There were 158 ESRD cases during follow-up. Incident ESRD rates were 178.8 per 100,000 person-years (10^5^ py) in high poverty outlier counties and were 76.3 /10^5^ py in affluent outlier counties, p trend = 0.06. In unadjusted competing risk models, persons residing in high poverty outlier counties had higher incidence of ESRD (which was not statistically significant) when compared to those persons residing in counties with neither high poverty nor affluence [hazard ratio (HR) 1.54, 95% Confidence Interval (CI) 0.75-3.20]. This association was markedly attenuated following adjustment for socio-demographic factors (age, sex, race, education, and income); HR 0.96, 95% CI 0.46-2.00. However, in the same adjusted model, income was independently associated with risk of ESRD [HR 3.75, 95% CI 1.62-8.64, comparing the < $20,000 income group to the > $75,000 group]. There were no statistically significant associations of county measures of poverty with incident ESRD, and no evidence of effect modification.

**Conclusions:**

In contrast to annual family income, geospatially-linked measures of county poverty have little relation with risk of ESRD. Efforts to mitigate socioeconomic disparities in kidney disease may be best appropriated at the individual level.

## Background

The risk of end-stage renal disease (ESRD) is increased among low income individuals and in low income communities [1]. Low income or poverty status is one of the most frequently studied indicators of low socioeconomic status (SES) at the individual level [2]. Poverty has been associated with multiple risk factors for kidney disease including hypertension [3], diabetes [4], and obesity [5]; and multiple studies have documented an association of poverty with kidney disease [6–10].

Community measures of SES have also been examined in relation to kidney disease. Volkova *et al.* reported that residence in poor U.S. neighborhoods in Georgia, North Carolina, or South Carolina, defined by the proportion of the census tract population living below the poverty level, were associated with higher rates of incident ESRD. [9] Grace *et al.* documented that incidence of renal replacement therapy was inversely associated with area advantage among non-indigenous Australian patients [11]. Upstream from ESRD, Merkin *et al.* found in two separate populations that living in a low SES area was independently associated with greater risk for progressive chronic kidney disease (CKD) [12, 13]. Additionally, Hossain *et al*. documented that area level SES was inversely correlated with rapid CKD progression among a hospital-based cohort in the U.K. [14]. We have also previously reported that household, but not community poverty, is associated with lower estimated glomerular filtration rate [8]. Additionally, residence in disadvantaged communities has been associated with other adverse health outcomes including impaired physical fitness [15], greater biological ‘wear and tear’ (as measured by allostatic load) [16], greater prevalence of hypertension [17], greater risk of coronary heart disease [18] and diabetes [4]; all of which can influence outcomes among persons with CKD.

A limitation of these prior analyses is that the associations of both community and individual SES with ESRD incidence were not examined, and it is possible that socioeconomic disparities may represent interactions between these two distinct influences [19, 20]. Understanding the associations of community and individual SES with kidney disease, and their potential interaction, could lead to more meaningful appropriation of resources aimed at the mitigation of disparities in CKD.

The objective of our study was to examine the independent associations of both individual and community SES with incident ESRD, focusing particularly on the concentration of poverty in communities. We hypothesized that residence in a poor community surrounded by other poor communities may pose a greater threat to an individual’s health than would residence in a poor community surrounded by more affluent communities. In the latter case, resources in the more affluent community may be accessible by those in the poor community. We examined these associations in the Reasons for Geographic and Racial Differences in Stroke (REGARDS) study.

## Methods

### Study design and population

REGARDS is a population-based cohort of black and white U.S. adults aged 45 years and older [21, 22]. A stratified random sample of eligible individuals was recruited from North Carolina, South Carolina, and Georgia, Alabama, Mississippi, Tennessee, Arkansas and Louisiana (56%), with the remaining 44% of the sample recruited from the other 40 contiguous U.S. states and the District of Columbia. Recruitment was from January 2003 to October 2007. Written consent was obtained from each participant. The institutional review boards (research ethics committees) of the participating institutions approved the study [21]. A full list of participating REGARDS investigators and institutions can be found at http://www.regardsstudy.org.

There were 30,183 REGARDS participants who completed an in-home examination. For this study, we excluded participants with addresses that could not be geocoded (due to their only providing post office boxes rather than street addresses; n = 6,333), those treated with dialysis at study enrollment (n = 116), and those missing follow up data within the study period (n = 420); final N = 23,314.

### Data collection and measures

Individual participant data were obtained during a telephone interview and subsequent in-home examination. During the interview, we ascertained participant’s age, sex, race, educational attainment, health insurance status, annual household income, and history of hypertension and/or diabetes. Home addresses used for the in-home visit were geocoded to the U.S. census tract level. Blood pressure was measured twice during the in-home visit with an aneroid sphygmomanometer following three minutes of sitting with both feet on the floor. The average of the two blood pressure measurements was used. Hypertension was defined as self-reported use of antihypertensive medications, a systolic blood pressure ≥140 mmHg, or a diastolic blood pressure ≥90 mmHg. Venous blood was collected for serum creatinine and glucose. Diabetes was defined by either self-report, prescribed oral hypoglycemic medications or insulin, fasting glucose ≥126 mg/dL or non-fasting glucose ≥200 mg/dL. Serum creatinine was measured by colorimetric reflectance spectrophotometry using the Ortho Vitros Clinical Chemistry System 950IRC instrument (Johnson & Johnson Clinical Diagnostics, Rochester, NY). The creatinine assay was calibrated to a creatinine standard determined by isotope dilution mass spectrometry [23]. Estimated glomerular filtration rate (eGFR) was calculated using the available single serum creatinine measurement for each participant, and the 4-variable estimating equation modified for the international calibration standards published by the Chronic Kidney Disease Epidemiology Collaboration [24].

Self-reported household income and the degree of concentrated poverty in the community of residence at the time of the in-home interview were used as individual and community level exposures in our hierarchical models described below. Annual household income for a participant was ascertained by asking “Is your annual household income from all sources less than…?”, and then specifying income levels from U.S. dollars (USD) <5,000 to USD >150,000 [25]. We grouped income levels into five categories: refused to provide, <$20,000; ≥$20,000 and < $35,000; ≥$35,000 and < $75,000; and ≥ $75,000.

The geocoded home address was used to assign a level of geographically concentrated county poverty using data obtained from the 2000 U.S. Census. The degree of poverty in a county was calculated by combining two county-level attributes: 1) a standardized Z score (Z = [county mean poverty level- mean poverty level across all counties]/ [standard deviation of county poverty across all counties]); and 2) the degree of local spatial autocorrelation of the Z score with the score of nearby counties [26]. The Z-score is a dimensionless measure of the deviation of a value from the overall group mean in units of the measure’s standard deviation. For example, a Z-score of 2.0 for any variable means that the value is 2 SD above and a Z-score of –2.0 is 2 SD below the overall mean for that measure and is comparable to a similar Z-score for any other measure. We defined concentrated poverty as counties with a Z-score greater than 2 above the mean poverty rate for U.S. counties and a local spatial autocorrelation (Moran’s score) Z-score greater than 2. In a similar fashion we categorized counties having concentrated affluence and those with neither concentrations of poverty or affluence. After inspection of the resulting distribution of counties on the U.S. map we defined the following categories of concentrated spatial wealth: outlier poverty, extremely high poverty, very high poverty, high poverty, neither, high affluence and outlier affluence [26]. Outlier counties are those that are more impoverished or affluent than would be expected given the level of poverty or affluence of the adjoining counties.

To confirm that geographically concentrated county poverty data from 2000 reflected more recent data, we examined community material disadvantage across county poverty levels using a measure developed and validated by Diez-Roux and colleagues [27]. Briefly, a neighborhood poverty score was calculated as the sum of the Z-scores of six measures of material well-being collected by the 2010 US Census at the census block level. The variables used in the construction of the neighborhood score included log of the median household income; log of the median value of housing units; the percentage of households receiving interest, dividend, or net rental income; the percentage of adults 25 years of age or older who had completed high school; the percentage of adults 25 years of age or older who had completed college; and the percentage of employed persons 16 years of age or older in executive, managerial, or professional specialty occupations. A summary score (neighborhood poverty Z-score) was defined as the sum of the six Z-scores. Additionally, we examined the Gini index, which is a measure of wealth inequality (range of 0-1, with 0 equal to complete equality, and 1 complete inequality) [28]. We also assessed the percentage of households in each county poverty category who were (a) living below poverty threshold, (b) female-headed, (c) with household income < $30,000, (d) without a vehicle, (e) vacant, (f) receiving public assistance, and (g) unemployed. Finally, we examined the rural urban commuting area (RUCA) codes for each participant’s zip code to determine whether they resided in a metropolitan (RUCA score 1-3), micropolitan (score 4-6) or rural (score 7-10) area [29].

Our outcome of interest was incident ESRD identified through linkage of REGARDS study participants with the United States Renal Data System (USRDS). The USRDS ESRD database is a national registry of patients receiving renal replacement therapy [30]. This analysis included incident ESRD cases through August 2009 defined as the first date of dialysis documented on the ESRD Medical Evidence Form of the Centers for Medicare and Medicaid Services (form CMS-2728) and recorded by the USRDS. Person-time was censored at ESRD, death, or date of last follow-up phone contact, whichever occurred first.

### Statistical analysis

We used ANOVA and chi-square tests to assess differences across county poverty category. We used multivariable Cox proportional hazards models to examine the independent association between income and county poverty measures and incident ESRD, while accounting for death as a competing risk using the Fine and Gray method [31]. We began with models that included interaction terms between individual income and county poverty. An interaction was assessed based on the statistical significance of these interaction terms. As none were statistically significant, our adjusted Model 1 included age, sex, race, and education. Model 2 also included income. The proportional hazards assumption was tested by examining the log-log survival plots-2 * log likelihood plots. Statistical analyses were performed using SAS version 9.2 (SAS Institute, Cary NC).

## Results

### Participant characteristics by county poverty category

Our study cohort consisted of 23,314 REGARDS participants (Table 1). Their mean age (SD) was 64.8 (9.4) years, 55.5% were female, and 41.0% were black. The majority of participants resided in the “stroke belt” (inland areas of North Carolina, South Carolina, and Georgia; as well as Alabama, Mississippi, Tennessee, Arkansas and Louisiana) and in metropolitan areas. Less than a high school education was reported by 12.5%. Few participants (6.7%) lacked health insurance. Household income was reported as less than $20,000 per year by 29.8%, $20-34,999 by 24.1%, $35,000 to 74,999 by 18.0%, greater than $75,000 by 12.5% and 15.5% refused to report their income.

**Table 1:** **REGARDS participant characteristics by county poverty level**

	All	Concentrated poverty				Neither	Affluence	
County poverty		High outlier	Extremely high	Very high	High		Low/Very low poverty	Low outlier poverty
	23,314	793 (3.4%)	786 (3.4%)	1,747 (7.5%)	4,198 (18.0%)	11,631 (49.9%)	2,147 (9.2%)	2,012 (8.6%)
**Demographics**								
Age in years (SD)	64.8 (9.4)	64.8 (9.3)	64.3 (9.4)	64.1 (9.5)	64.4 (9.4)	65.2 (9.5)	65.0 (9.4)	64.8 (9.3)
Female, %	55.5	57.1	53.3	58.2	58.0	56.2	48.1	51.7*
Black race, %	41.0	77.8	52.8	37.5	47.5	42.3	26.7	18.3*
Stroke belt resident, %	61.8	0	98.8	96.5	91.4	57.1	0	72.5*
Urban/Rural status, %								
Rural	9.3	0.8	33.6	41.1	7.6	5.5	8.0	2.7*
Micropolitan	13.4	0.9	34.1	41.8	14.2	9.5	7.4	13.2*
Metropolitan	77.2	98.4	32.3	17.1	78.2	85.0	84.6	84.1*
**Socioeconomic status**								
Education (%)								
Less than high school	12.5	18.1	18.1	18.4	16.1	11.1	8.5	8.2*
High school graduate	25.8	30.0	26.5	29.9	27.6	24.6	27.0	22.4
Some college	27.1	29.6	22.3	22.8	26.2	27.5	29.8	28.7
College graduate and above	34.6	22.4	33.2	28.9	30.0	36.9	34.7	40.7
No health insurance, %	6.7	6.7	10.9	9.2	8.3	6.3	4.7	4.2*
Annual household income, %								
<$20,000	18.0	24.1	26.1	24.5	21.6	16.7	13.5	11.9*
$20,000 to <35,000	24.1	28.2	24.4	24.8	25.2	23.5	25.2	21.7*
$35,000 to <75,000	29.8	24.6	24.7	26.5	28.8	30.4	32.4	33.2*
≥$75,000	15.5	10.1	10.9	12.9	11.7	16.6	17.2	21.5*
Refused to provide income	12.5	13.0	13.9	11.2	12.7	12.8	11.8	11.7*
**Clinical factors**								
Estimated GFR (CKD-EPI) (SD)	85.1 (19.9)	87.1 (22.8)	87.3 (20.5)	85.3 (20.2)	86.5 (20.3)	84.6 (19.9)	84.2 (18.7)	84.7 (18.1)*
Diabetes mellitus, %	22.0	23.7	26.4	26.3	25.2	21.6	16.4	17.4*
Hypertension, %	59.4	69.2	64.1	62.8	64.4	58.1	53.9	53.4*

*Note:* Categories of county poverty were defined as: high outlier poverty, extremely high poverty, very high poverty, high poverty, neither, high affluence (low/very low poverty) and outlier affluence (low outlier poverty) [26]. Outlier counties are those that are more impoverished or affluent than would be expected given the level of poverty or affluence of the adjoining counties. The “stroke belt” includes inland areas of North Carolina, South Carolina, and Georgia; as well as Alabama, Mississippi, Tennessee, Arkansas and Louisiana.

*p < 0.05, chi-square test or ANOVA method.

*Abbreviations:* *SD* standard deviation.

Nearly half (49.9%) of respondents lived in counties where there was neither concentrated poverty nor affluence and 32.8% lived in counties with varying degrees of concentrated poverty (mean county household income 2 or more standard deviations below the national mean that was correlated with contiguous and nearby counties) (Table 1). Individuals living in counties characterized by greater concentrated poverty were more likely to be female, of black race and with less than a high school education. As was expected from the original description of the concentrated poverty counties by Holt [26] nearly all of the respondents living in counties with concentrated poverty were residents of the U.S. “stroke belt” and these individuals were more likely to live in rural areas than those residing in affluent counties. In contrast, none of the respondents living in outlier poverty counties were “stroke belt” residents and nearly all (98.4%) resided in metropolitan areas.

The association between reported household income and concentrated county poverty was as expected. Incomes of less than $20,000 were more frequently reported by individuals living in the most concentrated poverty. There was no clear pattern for incomes in the $20,000 to $34,999 category, while participants with incomes > $35,000 or > $75,000 comprised a greater proportion of those residing in more affluent counties (Table 1). For example, 10.9% of individuals living in counties in the extremely concentrated poverty category reported personal household incomes of $75,000 or greater compared to 17.2% of individuals in the more affluent category. Participants who refused to report household income comprised a greater proportion of those residing in concentrated poverty counties than those in the affluent counties.

Several clinical factors varied across county poverty categories. Individuals living in concentrated poverty counties had a greater prevalence of diabetes and hypertension than those in affluent counties; and estimated GFR was slightly higher among persons in concentrated poverty counties.

### County characteristics by county poverty category

Measures of material disadvantage were associated with a greater degree of concentrated county poverty (Table 2). The proportion of households with incomes below the U.S. federal poverty line, headed by females, with incomes less than $30,000 per year, reporting no automobiles available to the household, vacant housing, on public assistance and unemployed were all higher among counties with concentrated poverty (Table 2). Median household incomes ranged from $30,905 in counties with the highest degree of concentrated poverty to $53,279 in affluent counties. The Gini index, a measure of income inequality within a county, with higher values indicative of greater inequality [28], were slightly higher in poor counties.

**Table 2:** **County characteristics by county poverty levels (based on 2010 U.S. census data)**

	Concentrated poverty				Neither	Affluence	
Characteristic	High outlier	Extremely high	Very high	High		Low/Very low poverty	Low outlier poverty
Neighborhood poverty Z-scores	-2.07	-3.26	-3.06	-1.64	0.78	1.82	2.21
Mean Gini index	0.47	0.51	0.47	0.48	0.46	0.44	0.44
Households below poverty (%)	21.4	29.1	24.8	19.7	15.6	12.2	11.6
Female-headed household (%)	30.8	34.6	29.2	27.7	22.2	17.7	17.2
Median income	$41,449	$30,950	$33,407	$41,375	$49,005	$53,279	$54,725
Household income < $30,000 (%)	30.9	42.0	38.6	31.1	25.6	22.0	20.9
No vehicle (%)	13.4	14.4	10.1	8.6	8.2	6.5	4.6
Vacant housing (%)	15.4	21.2	16.2	14.3	12.3	9.9	13.5
Public assistance (%)	18.3	22.9	19.2	15.4	10.6	9.7	8.4
Unemployed (%)	12.1	10.3	8.7	7.1	7.2	6.5	6.1

*Note:* P-values for all rows were <0.05. Categories of county poverty were defined as: high outlier poverty, extremely high poverty, very high poverty, high poverty, neither, high affluence (low/very low poverty) and outlier affluence (low outlier poverty) [26]. Outlier counties are those that are more impoverished or affluent than would be expected given the level of poverty or affluence of the adjoining counties. The neighborhood poverty score was calculated as the sum of the Z-scores of six measures of material well-being collected by the 2010 U.S. Census at the census block level. Higher values indicate greater neighborhood poverty. The Gini index is a measure of income inequality within a county, with higher values indicative of greater inequality.

### Incident ESRD by county poverty category

**Figure 1:**
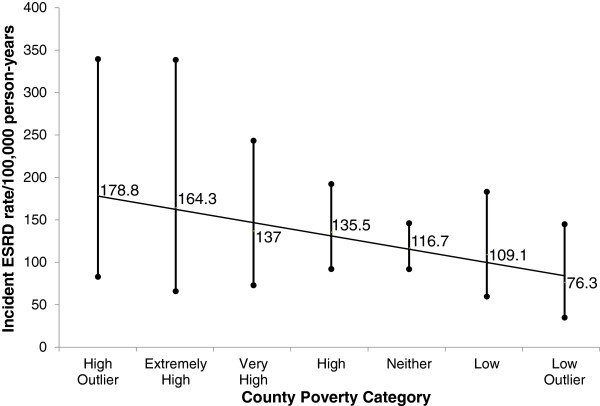
**Incident ESRD rate/100,000 person years (95% confidence interval) by County Poverty Category.** There were 158 ESRD cases during follow-up. Incident ESRD rates declined from 178.8 per 100,000 person-years (10^5^ py) in high poverty outlier counties to 76.3 /10^5^ py in affluent outlier counties, p trend = 0.06.

Median follow up for our study was 6.0 years, and a total of 2,615 participants died during follow up. There were 158 incident cases of ESRD of follow up. Incident ESRD rates were 178.8 per 100,000 person-years (10^5^ py) (95% confidence interval [CI], 83.0 to 339.5/10^5^ py) among individuals in high poverty outlier counties and were 76.3 /10^5^ py (95% CI 34.9 to 144.9/10^5^ py) in affluent outlier counties, p for trend = 0.06 (Figure 1). In unadjusted competing risk models, persons residing in high poverty outlier counties had higher incidence of ESRD (which was not statistically significant) when compared to those persons residing in counties with neither high poverty nor affluence [hazard ratio (HR) 1.54, 95% Confidence Interval (CI) 0.75-3.20]. This association was markedly attenuated following adjustment for socio-demographic factors (age, sex, race, education, and income); HR 0.96, 95% CI 0.46-2.00. In this multivariable competing risk model, only income was independently associated with risk of ESRD [hazard ratio (HR) 3.75, 95% confidence interval (CI) (1.62-8.64) comparing the < $20,000 group to the > =$75,000 group] (Table 3). There were no statistically significant associations of county poverty with incident ESRD. Furthermore, there was no statistically significant interaction between income and county poverty category. We did not perform further adjustment of our models for other factors noted in Table 1, such as baseline kidney function, diabetes and hypertension status, out of concern for potentially inducing biased estimates [32].

**Table 3:** **Hazard ratios for incident ESRD by county poverty and individual household income accounting for the competing risk of mortality**

	Unadjusted	Model 1*	Model 2**
	n = 23,314	n = 23,295	n = 23,295
**ESRD, total events = 158**			
**County poverty levels (number of ESRD events)**			
High outliers (8)	1.54 (0.75-3.20)	1.03 (0.50-2.13)	0.96 (0.46-2.00)
Extremely high (7)	1.36 (0.63-2.95)	1.20 (0.55-2.61)	1.10 (0.51--2.39)
Very high (13)	1.14 (0.63-2.05)	1.25 (0.69-2.28)	1.16 (0.64-2.10)
High (31)	1.13 (0.74-1.72)	1.09 (0.71-1.66)	1.04 (0.68-1.58)
Neither (76)	Ref	Ref	Ref
Low and very low (14)	1.00 (0.56-1.76)	1.21 (0.69-2.14)	1.19 (0.68-2.10)
Low outliers (9)	0.68 (0.34-1.36)	1.00 (0.49-2.04)	1.01 (0.50-2.06)
**Demographics**			
Age, years, continuous		1.02 (1.01-1.04)	1.02 (1.00-1.03)
Male		1.63 (1.19-2.22)	1.82 (1.34-2.49)
Black (compared to white) race		4.18 (2.89-6.06)	3.59 (2.47-5.23)
**Socioeconomic status**			
Education < High School (H.S.) compared to > =H.S.		1.18 (0.78, 1.78)	0.98 (0.64-1.51)
Income category, %			
Refused			2.17 (0.87-5.43)
<$20,000			3.75 (1.62-8.64)
$20,000 to <35,000			4.55 (2.04-10.13)
$35,000 to <75,000			1.56 (0.66-3.64)
≥$75,000			Ref

*Adjusted for county poverty, age, sex, race and education.

**Adjusted for county poverty, age, sex, race, education and income.

## Discussion

Among a population-based cohort of U.S. adults, we found that socio-demographic factors, including household income, varied in an expected direction across county poverty levels, and the county poverty metric clearly defined levels of material disadvantage for this population. We report that the well-known association of low income with increased risk of ESRD is strong and independent of geospatially-linked measures of county poverty.

There are several potential explanations for our findings. First, we found that REGARDS participant characteristics well-known to be associated with poverty and CKD varied in an expected direction across geospatially-linked measures of county poverty. We also found a non-statistically significant association between high poverty outlier counties and higher incidence of ESRD in our unadjusted analyses (Figure 1). However, the county poverty association was almost entirely accounted for by differences in race and income (Table 3). Thus, constitutional factors (e.g. genetic background) and individual income may be far more potent predictors of ESRD than community SES. Second, the well-established and plausible association of community SES and health outcomes may vary for different patient populations. For example, in contrast to our findings, Merkin *et al.* reported that among male and female adults 65 years and older, residence in a low SES area was associated with progressive CKD, however individual level income was not [13]. Notably, Merkin *et al*. found in a younger population that residence in a low SES area was independently associated with progressive CKD only among white men, with no significant associations documented for white women or African Americans [12]. Their findings also raise a third potential explanation for our study findings, which is that individual versus community resources may play different roles in different stages of CKD. One could envisage, for example, that community resources may play a greater role in early development of CKD via their influence on the development and management of key risk factors for CKD (e.g. type 2 diabetes that develops as a consequence of limited physical activity). However, a recent study by Gaskin *et al.* found that household poverty status, but not neighborhood poverty concentration, was independently associated with odds of having diabetes [33]. Thus individual income, as compared to community SES, may be a more important determinant of CKD risk factors as well as CKD progression.

Our findings have important implications. The Healthy People 2020 initiative, the U.S. national blueprint for public health goals, aims to eliminate socioeconomic health disparities among patients with kidney disease in the U.S. by 2020 [34]. Achieving this goal will require the identification of the root cause of socioeconomic disparities in CKD, as well as targeted public and clinical interventions to mitigate these disparities. The findings of our study support a focus on individual rather than community resources when attempting to reduce disparities in ESRD, and emphasize the need to prevent and better manage established CKD risk factors such as diabetes and hypertension among low income individuals.

## Conclusions

Our study had limitations. First, not all participants’ residences could be geocoded and many did not provide information about their annual household income. Those who did not provide their incomes comprised a greater proportion of the participants residing in less affluent communities than they did those living in more affluent communities. We also found a non-statistically significant trend towards greater risk of ESRD in this population, compared to those reporting earning at least $75,000/year. Second, our measure of annual household income did not take into account household size and therefore may have failed to classify individuals with larger household income and a large family as economically disadvantaged. Therefore, our estimates of the relation between income and ESRD are conservative. Third, the small number of ESRD events in certain county poverty categories may have limited our power to detect statistically significant associations. Fourth, we lacked a measure of SES across the life course [35] and participants may have changed residence (and potentially, county poverty category) during the follow-up period. Additionally, it is possible that advanced kidney disease could have led to economic disadvantage over the life course for some participants (ie. reverse causality). Fifth, the majority of participants in our study resided in the U.S. ‘stroke belt’, thus our results may not be generalizable to other U.S. population samples. While we found that income was strongly and independently associated with risk of ESRD, an analysis of the Kidney Early Evaluation Program, a nationwide community-based screening study of persons at high risk of kidney disease, found that other measures of individual SES (education and health insurance) were not independently associated with incident ESRD [36]. Finally, and importantly, counties may not be the optimal proxies for geography (e.g. they may be too large of an area) and our analysis is subject to potential bias due to the modifiable areal unit problem [37]. Neighborhood, census tract or ZIP code level poverty measures may have yielded different findings in our study. The limitations of our study are balanced by its large number of participants with well-balanced representation of white and black adults, and this being the first study to examine the relative associations of individual and community SES with incident ESRD.

In conclusion, as compared to annual family income, geospatially-linked measures of community poverty have little relation with risk of ESRD. Efforts to mitigate socioeconomic disparities in CKD may be best appropriated at the individual level.
